# Integrated Analysis of Multiscale Large-Scale Biological Data for Investigating Human Disease

**DOI:** 10.1155/2015/760765

**Published:** 2015-04-20

**Authors:** Tao Huang, Lei Chen, Mingyue Zheng, Jiangning Song

**Affiliations:** ^1^Mount Sinai School of Medicine, New York, NY 10029, USA; ^2^Shanghai Maritime University, Shanghai 201306, China; ^3^Chinese Academy of Sciences, Shanghai 201203, China; ^4^Monash University, Clayton, VIC 3800, Australia

In recent years, more and more omics data is generated. Even for the same samples, multiple levels of omics data can be measured in large scale. These multiscale and large-scale data could help in revealing the biological basis of complex diseases and optimizing the therapeutic strategies. Analysis of such data is very challenging since the data is inaccessible in the past and few methods are developed. In this special issue, we presented 17 novel studies about the analysis method of such complex data and their applications to interesting medical and biological questions.

Y. Jiang et al. proposed a method to identify novel thyroid cancer-related genes and chemicals using shortest path algorithm. Some of the identified genes are crucial to the tumorigenesis and development of thyroid cancer. This method can be generalized to identify genes for other complex diseases.

W. Kong et al. constructed the common and brain region specific subnetworks of Alzheimer's disease. The identified common subnetworks across six brain regions suggested that inflammation of the brain nerves is one of the critical factors of Alzheimer's disease and calcium imbalance may link several causative factors of Alzheimer's disease.

M. F. Rogers et al. studied supervised interactive network inference using multiple kernel learning. The proposed method was composed of cautious classification and data cleaning, where cautious classification was used to increase the accuracy by restricting predictions to high-confidence instances, whereas data cleaning was used to mitigate the influence of mislabeled training instances.

J. Che and M. Shin proposed a meta-analysis strategy for gene prioritization that integratively employs three different genetic resources: gene expression data, single nucleotide polymorphism (SNP) genotype data, and expression quantitative trait loci (eQTL) data. The strategy for gene prioritization showed its superiority to conventional methods in discovering significant disease-related genes with several types of resources, while making good use of potential complementarities among available genetic resources.

J. E. Vargas et al. used a network flow approach to predict protein targets and flavonoid backbones to treat respiratory syncytial virus (RSV) infection. They identified 26 flavonoids and 5 compounds through topological analysis of chemical-protein and protein-protein interaction network. Some mechanisms of action of early RSV infection were uncovered.

Y. Dong et al. reported a support vector machine (SVM) model for predicting new interactions between the human papillomavirus 16 (HPV16) and other proteins. The analysis of protein-protein interactions indicated that HPV16 enlarged its scope of influence by interacting with human proteins as much as possible, and these interactions alter a broad array of cell cycle progression.

Y. Zhang et al. proposed a computational approach, integrating Ping-Pong algorithm and multiobjective genetic algorithm, to identify subtype-specific miRNA-mRNA functional regulatory modules. And this method was applied to investigate subtype-specific miRNA-mRNA functional regulatory modules of multiple myeloma.

D. Li et al. identified novel breast cancer subtype-specific biomarkers by integrating genomics analysis of DNA copy number aberrations and miRNA-mRNA dual expression profiling. The predicted correlation between BRCA1 and miR-143/miR-145 was validated by experiments and miR-143/miR-145 were promising novel biomarkers for breast cancer subtyping.

Z.-H. You et al. proposed a method to detect large-scale protein-protein interactions by integrating big biosensing data with computational model. The model was based on extreme learning machine (ELM) combined with a novel representation of protein sequence descriptor. The accuracy of their method was 84.8% while the sensitivity and specificity were 84.08% and 85.53%, respectively. It outperformed support vector machine (SVM).

J. Zhang et al. developed an effective method for distinguishing age-related macular degeneration (AMD) related genes using gene ontology (GO) and KEGG enrichment scores. 720 GO terms and 11 KEGG pathways were found to be important for predicting AMD related genes. These GO terms and KEGG pathways could help understand the underlying mechanisms of AMD.

X. Li et al. conducted a genome-wide association study of CNVs in two large-scale lung cancer datasets: the Environment And Genetics in Lung cancer Etiology (EAGLE) and the Prostate, Lung, Colorectal and Ovarian (PLCO) Cancer Screening Trial datasets. With a combined analysis of the association accordance between the two datasets, they identified 167 risk SNP loci and 22 CNVs associated with lung cancer and linked them with recombination hotspots.

X. Wang et al. evaluated the use of the KDIGO staging system for the prediction of prognosis in patients with septic acute kidney injury in intensive care units in Beijing, China, via a multicenter clinical study. Six independent risk factors for mortality were identified, which may help in making early and accurate diagnosis and adopting preventive and therapeutic interventions that could reduce mortality rates in the patients.

Y.-F. Gao et al. proposed a graphic method to identify novel glioma related genes. The known glioma related genes were mapped onto a weighted protein-protein interaction network and the genes that link the known glioma related genes on the network were considered as candidate novel genes. The candidate genes were further filtered by permutation test. Some of the final novel glioma related genes were supported by latest literatures.

L. He et al. combined the statistical algorithm with the gene-pathway bipartite networks to generate the reliable lists of cancer-related DEGs and constructed the models, which can be used for predicting the prognosis of three types of cancers, namely, breast cancer, acute myeloma leukemia, and glioblastoma.

L. Zhu et al. investigated the genome-wide gene expression in* Gleditsia sinensis* with transcriptome sequencing which generated 58583 unigenes. In these genes, 31385 unigenes were annotated. What is more, a PPI network was constructed and used to predict new stress resistance genes, in order to provide a platform for future functional genomic studies.

N. Zhang et al. proposed a computational method to identify influenza A/H7N9 virus infection-related human genes from shortest paths in a virus-human protein interaction network. Finally, 20 human genes were screened out which could be the most significant, providing guidelines for further experimental validation.

L. Guo et al. performed an integrated analysis of miRNA, lncRNA, and mRNA expression profiles in human HepG2 and L02 cells. They found that isomiR repertoires and expression patterns might contribute to tumorigenesis through different biological roles. The cross-talk between different RNA molecules could help reveal the complex mechanisms underlying tumorigenesis.

How to analyze the multiscale large-scale biological data is one of the most important questions in postgenomic era. It is even more important than generating these data. It is the battlefield for computational scientists, the bridge between biotechnology and clinical applications, and the stepping stone for translational medicine. The collaborations between scientists from different backgrounds are essential since no one can fully understand and completely solve such complex question by himself. The complexities of biological system amaze people and inspire people to investigate with all means.

*Tao Huang* *Lei Chen* *Mingyue Zheng* *Jiangning Song*

